# Screening drug target combinations in disease-related molecular networks

**DOI:** 10.1186/s12859-019-2730-8

**Published:** 2019-05-01

**Authors:** Min Luo, Jianfeng Jiao, Ruiqi Wang

**Affiliations:** Department of Mathematics, Shanghai University, No.99, Shangda Road, Shanghai, China

**Keywords:** Drug target combination, Disease network, Combination index, Biology system

## Abstract

**Background:**

For treating a complex disease such as cancer, some effective means are needed to control biological networks that underlies the disease. The one-target one-drug paradigm has been the dominating drug discovery approach in the past decades. Compared to single target-based drugs, combination drug targets may overcome many limitations of single drug target and achieve a more effective and safer control of the disease. Most of existing combination drug targets are developed based on clinical experience or text-and-trial strategy, which cannot provide theoretical guidelines for designing and screening effective drug combinations. Therefore, systematic identification of multiple drug targets and optimal intervention strategy needs to be developed.

**Results:**

We developed a strategy to screen the synergistic combinations of two drug targets in disease networks based on the classification of single drug targets. The method tried to identify the sensitivity of single intervention and then the combination of multiple interventions that can restore the disease network to a desired normal state. In our strategy of screening drug target combinations, we first classified all drug targets into sensitive and insensitive single drug targets. Then, we identified the synergistic and antagonistic of drug target combinations, including the combinations of sensitive drug targets, the combinations of insensitive drug target and the combination of sensitive and insensitive targets. Finally, we applied our strategy to Arachidonic Acid (AA) metabolic network and found 18 pairs of synergistic drug target combinations, five of which have been proven to be viable by biological or medical experiments.

**Conclusions:**

Different from traditional methods for judging drug synergy and antagonism, we propose the framework of how to enhance the efficiency by perturbing two sensitive targets in a combinatorial way, how to decrease the drug dose and therefore its side effect and cost by perturbing combinatorially a main sensitive target and an auxiliary insensitive target, and how to perturb two insensitive targets to realize the transition from a disease state to a healthy one which cannot be realized by perturbing each insensitive target alone. Although the idea is mainly applied to an AA metabolic network, the strategy holds for more general molecular networks such as combinatorial regulation in gene regulatory networks.

**Electronic supplementary material:**

The online version of this article (10.1186/s12859-019-2730-8) contains supplementary material, which is available to authorized users.

## Background

In the past decades, effective treatments of a complex diseases require practical means to control the biological networks underlying the disease. Biological networks are often robust to external disturbances, so it is difficult to control network dynamics by controlling a single target, and drugs targeting multiple targets may counter these troubles [1]. It has been long realized that the behavior of drug molecules in a disease network can be complex. The one-target one-drug paradigm has been the dominating drug discovery approach which result in many drugs marketed but cannot treat certain complex diseases sufficiently [2–4]. In fact, drug combination therapeutics are often more effective and are used to treat various complex diseases in recent years. To overcome the limitations of the single-target-based drugs, growing attention has been paid to drug discoveries involving multiple targets at the level of disease networks [5]. Actually, it has been a long history of using combination drugs to treat diseases. For example, the Traditional Chinese Medicines (TCM), especially herbal medicines, which can be viewed as the combinations of multiple compounds with synergy effects, have been used for thousands of years [6].

A diverse range of works have been carried out in the emerging field of multi-target drug design, many methods have been proposed to identify effective drug combinations [5, 7–11]. It is recommended that system-oriented drug design should consider the intrinsic properties of biological systems, such as robustness [12]. Many mathematical models of disease-relevant pathways have been constructed which have the potential to elucidate underlying mechanisms of diseases and to identify treatment strategies [13, 14]. And analyzing the properties of networks can attribute to identify potential drug targets and understand the connectivity between them [15, 16].

There may be many beneficial outcomes of synergy, such as increasing the therapeutic effect, reducing the dose but increasing or maintaining the same efficacy to avoid toxicity or reducing the cost of the drug, minimizing or slowing the development of drug resistance. For these therapeutic benefits, drug combinations have been widely used and become the leading choice for treating the most dreadful diseases, such as cancers and infectious diseases.

In this paper, we developed a strategy to screen multiply drug targets combinations. For a given disease network, we tried to identify effective points and the combination of interventions that can restore the disease network to a desired normal state. In our strategy, we first classified all drug targets into sensitive and insensitive single drug targets. Then, we identified synergy and antagonism for all drug target combinations which include the combinations of sensitive drug targets, the combinations of insensitive drug targets, and the combinations of sensitive and insensitive drug targets. We applied our strategy to the arachidonic acid (AA) metabolic network and we found 18 pairs of synergistic drug target combinations, five of which have been proven to be viable through biological or medical experiments.

## Results

## The statement of screening drug target combinations

Feedback loops, cross-talk and other network-intrinsic properties can make the effects of drug molecules much more complicated than predicted by a linear one-drug one-target approach [17]. We here develop a strategy to screen synergetic drug target combinations with pre-determined therapeutic effects. For convenience, two network states are defined in the disease network: the disease state and the normal state (or desired state). The disease state is a network state which the production of disease-related molecules is abnormal. The normal state is the network state which one would like to achieve after taking medicine. The main procedure of screening the synergetic drug target combinations is to perturb the network and optimize it toward the normal state.

### Determining the sensitive and insensitive individual drug targets

Before screening the synergetic drug target combinations, we need to identify the sensitive and insensitive individual drug targets. All single drug targets are ranked according to a criterion that measures their potential in restoring the network state when they are perturbed. In other words, we can rank the individual drug targets according to their sensitivities. The process of determining the sensitive and insensitive individual drug targets includes the following steps:

Step 1: Define the disease and normal states. A state can be defined as a steady state or transient of a network, which can be a collection of concentrations of proteins or metabolites. Generally, the disease state is a steady state of a disease network under standard parameters. The normal state is the desired state after perturbations.

Step 2: Select reactions that can be controlled by drugs. For example, if the drug targets are enzymes, conveniently, we mark all drug targets with *d*_1_,⋯,*d*_*i*_,⋯,*d*_*n*_, and mark the initial concentration of the *i*-th enzyme with *E*_*di*_.

Step 3: Change the concentration of the selected target enzyme until the network is translated into the normal state. We use *E*_*hi*_ to mark the concentration of the *i*-th enzyme after changing.

Step 4: Define the degree of change in concentration of each enzyme as △*E*_*i*_/*E*_*di*_, where △*E*_*i*_=|*E*_*di*_−*E*_*hi*_|. Rank the value of △*E*_*i*_/*E*_*di*_ from small to large. Then, we can have an order of drug targets, as shown in Table 1.

**Table 1:** Ranking the value of $\frac {\vartriangle E_{i}}{E_{di}}$ from small to large

Number	Enzyme	△*E*_*i*_/*E*_*di*_
1	*d*_1_	△*E*_1_/*E*_*d*1_<1
⋮	⋮	⋮
*j*	*d*_*j*_	△*E*_*j*_/*E*_*dj*_≤1
⋮	⋮	⋮
*n*	*d*_*j*_	△*E*_*j*_/*E*_*dj*_>1

We define a threshold of *a* and *a* is a constant. An enzyme which satisfies the condition △*E*_*i*_/*E*_*di*_≤*a* is defined as a sensitive drug target, and an insensitive drug target otherwise.

### Screening synergistic drug target combinations

#### Identifying the synergistic combinations of sensitive drug targets

With the development of medicine science and pharmacology industry, combinatorial drugs are becoming the standard to cure many complex diseases [18]. As a result, some methods have been proposed to identify effective drug combinations. Combination index (CI) analysis is widely used to evaluate drug interactions in combination drug disease treatment. The Loewe additivity model has been widely used when the combined effect of two drugs is additive. The model can be written as:

1 $$ \frac{(D)_{1}}{(Dx)_{1}}+\frac{(D)_{2}}{(Dx)_{2}}=1,  $$

where (*D*)_1_ and (*D*)_2_ are the respective combinatorial doses of drug 1 and drug 2, and (*Dx*)_1_ and (*Dx*)_2_ are the corresponding single doses for drug 1 and drug 2 with the same effect. When (1) holds, it can be concluded that the combinatorial effect of the two drugs is additive. Based on (1), the CI can be defined as:

2 $$ CI=\frac{(D)_{1}}{(Dx)_{1}}+\frac{(D)_{2}}{(Dx)_{2}}.  $$

According to (2), the CI can be used to classify drug interactions as synergistic, additive, antagonistic and hybrid [19]. And the corresponding curves are shown in [20].

1. Synergy: *CI*<1. In this case, the effect of the drug combination is superior to each single drug, for example, the combined drugs allow each drug to have a smaller dose. Therefore, the synergistic combination of drugs can exhibit fewer side effects in the treatment of diseases.

2. Antagonism: *CI*>1. In this case, the effect of each single drug is superior to the drug combination, and the combined drugs may require each drug to have more dose. This may lead to increased drug costs and greater side effects.

3. Additivity: *CI*=1. In this case, the efficiency of the combinatorial drugs is equal to the efficiency of the single drug. At the same time, this additivity type also provides a standard for judging synergistic and antagonistic combinations.

4. Hybrid. For a drug combination, some drug dose combinations $\left (\frac {(D)_{1}}{(Dx)_{1}}',\frac {(D)_{2}}{(Dx)_{2}}'\right)$ satisfy *CI*>1, while other drug dose combinations $\left (\frac {(D)_{1}}{(Dx)_{1}}'',\frac {(D)_{2}}{(Dx)_{2}}''\right)$ satisfy *CI*<1. That is to say, this drug combination can have synergistic or antagonistic effects, depending the drug doses of the combination.

In the past, Chou and co-workers have proposed semiquantitative methods for describing the degrees of synergism or antagonism [6]. These methods are now expanded as shown in Table 2 [21]. Using the CI grading, synergism is subdivided into nearly additive, slight synergism, moderate synergism, synergism, strong synergism, and very strong synergism and antagonism is divided by a similar way [21]. We mainly concern about the qualitative shape of the isobolograms for correctly identifying the drug pair categories, and use the smallest or largest CI of all drug dose combinations as the CI for this drug pair [22].

**Table 2:** Description and symbols of synergism or antagonism in drug target combination studies analyzed with the CI method

Range of Combination Index	Description	Graded Symbols
<0.1	very strong synergism	+ + + + +
0.1-0.3	strong synergism	+ + + +
0.3-0.7	synergism	+ + +
0.7-0.85	moderate synergism	+ +
0.85-0.90	slight synergism	+
0.90-1.10	nearly additive	+ -
1.10-1.20	slight antagonism	-
1.20-1.45	moderate antagonism	- -
1.45-3.3	antagonism	- - -
3.3-10	strong antagonism	- - - -
>10	very strong antagonism	- - - - -

In recent years, the development of disease types is very rapid, but the development of drugs is far slow than the development of diseases which leads to the case that there are no suitable drugs to cure many complex diseases. It is very difficult to find a new drug, which inspires us to find new combination of old drugs. Based on this biological significance, we try to study the combinations of sensitive drug targets.

For all drug target combinations of sensitive drug targets, we analyze the synergy and antagonism of the sensitive drug target combination by computing CI according to the doses of drugs. What’s more, we rank the degrees of synergism or antagonism of all drug target combinations according to Table 2.

#### Identifying the synergistic combinations of insensitive targets

In biomedical, when the two drugs are used alone, they can not cure a disease, but when the two drugs are used in combination, the disease can be cured or the effect can be obviously improved. The combination of insensitive drug targets may provided an idea for solving such problems.

For the insensitive drug targets, we combine them in pairs and calculate △*E*_*i*_/*E*_*di*_ where △*E*_*i*_=|*E*_*di*_−*E*_*hi*_|. The drug combinations which △*E*_*i*_/*E*_*di*_≤1 needs to be satisfied for the perturbation to insensitive targets can guarantee that the doses of drugs are used less than those used before combination. So, if each drug target of a combination satisfy △*E*_*i*_/*E*_*di*_≤1, then this combination is defined as synergistic combinations.

#### Identifying the synergistic combinations of sensitive and insensitive drug targets

In clinical treatment, many diseases do not have a suitable treatment because the used drugs are relatively expensive. If the dosage of expensive drugs can be reduced with the auxiliary of cheap drugs, a better treatment strategy is able to be designed. Based on the above ideas, the combinations of sensitive and insensitive drug targets may provide a feasible method.

For the combination of the sensitive and insensitive drug targets, we calculate the △*E*_*i*_/*E*_*di*_ of sensitive and insensitive drug targets respectively.

For the sensitive drug target, we define a constant *α* and set the following formula:

3 $$ \frac{{\left(\frac{\triangle E_{i}}{E_{di}}\right)}^{1}}{{\left(\frac{\triangle E_{i}}{E_{di}} \right)}^{2}}=\frac{{(\triangle E_{i})}^{1}}{({\triangle E_{i}})^{2}} = \alpha   $$

where (△*E*_*i*_/*E*_*di*_)^1^ is the value of △*E*_*i*_/*E*_*di*_ after combination and (△*E*_*i*_/*E*_*di*_)^2^ is the value of △*E*_*i*_/*E*_*di*_ before combination. *α* can be determined in advance and satisfy 0<*α*<1.

Then, we find the drug target combination which insensitive drug targets satisfy △*E*_*i*_/*E*_*di*_≤1 and the sensitive drug targets is (△*E*_*i*_)^1^/(△*E*_*i*_)^2^=*α*. Such combinations are defined as synergistic.

The principle of synergistic combinations of sensitive and insensitive drug targets is reducing the dosage of drugs perturbed to sensitive target by the auxiliary drug, i.e., the perturbation to insensitive drug target. The drug combinations in which △*E*_*i*_/*E*_*di*_≤1 needs to be satisfied for the perturbation to insensitive targets and (△*E*_*j*_)^1^/(△*E*_*j*_)^2^=*α*(0<*α*<1) for the sensitive target can guarantee that the doses of drugs are used less than those used before combination. In this case, the drug combinations can improve the efficacy and reduce the doses of drugs and their side effects. For example, for a combination of sensitive and insensitive drug targets, if the perturbation to the sensitive drug target satisfies (△*E*_*j*_)^1^≪(△*E*_*j*_)^2^, and the cost of drug for the insensitive target is relatively low, then such a perturbation combination may have more advantages.

## Applying the strategy of screening drug target combinations to AA metabolic network

### The metabolic network of AA in human PMNs

**Fig. 1:**
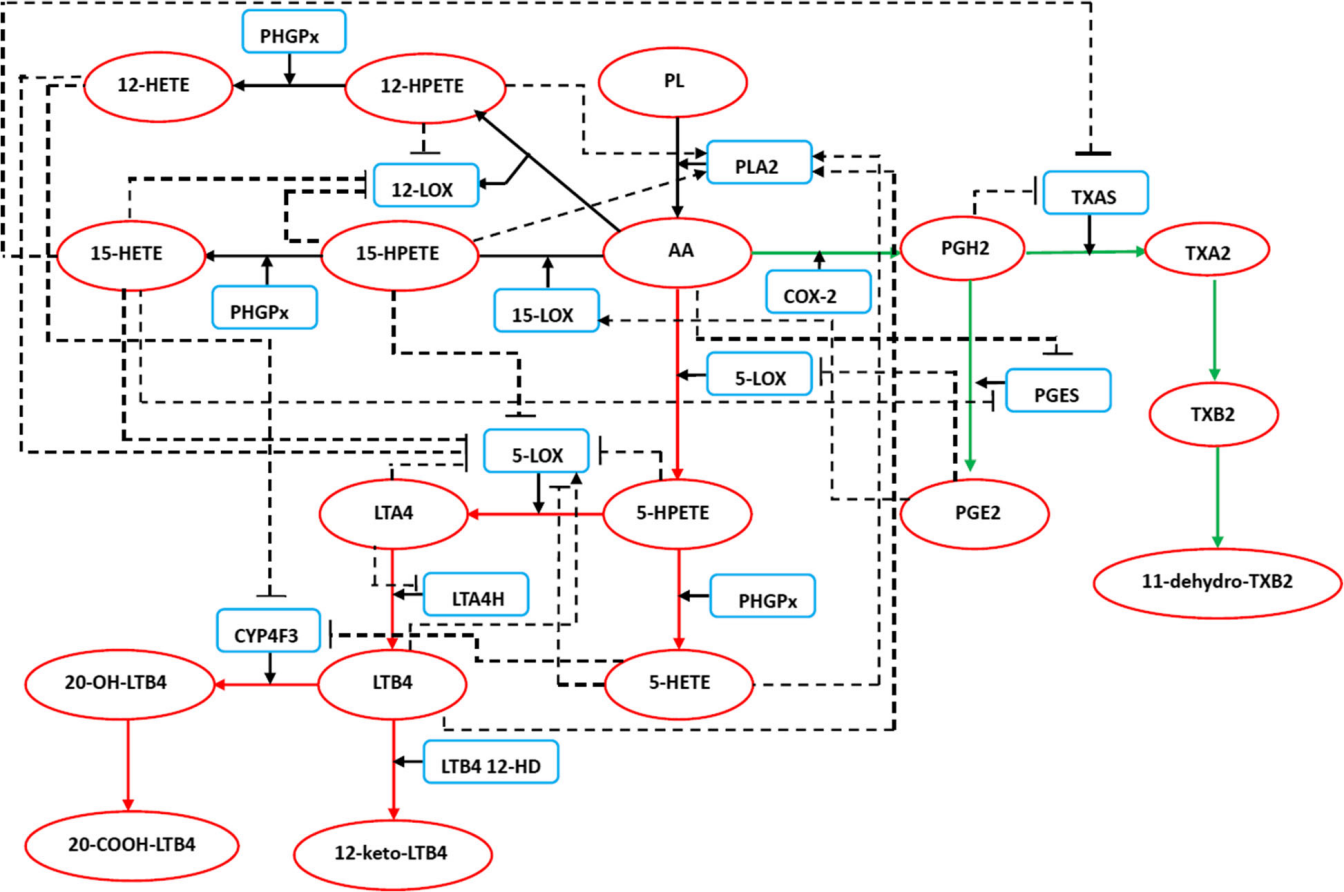
The AA metabolic network in human PMNs. AA is metabolized through three main pathways: the 5-LOX pathway (red line), the 15-LOX pathway (blue line), and the COX-2 pathway (green line). PGE2, LTA4, and LTB4 are the major inflammatory mediators produced in the COX-2 and 5-LOX pathways. A total of 24 feedback loops, which are involved in the network, make important contributions to the regulation of inflammatory mediators

Now, we apply our strategy to the Arachidonic Acid (AA) metabolic network. There have been some studies on drug combinations of AA metabolic network [23]. Inflammation is a type of nonspecific immune response to infection, irritation, or other injury. It is characterized by redness, swelling, pain, and some loss of function [24]. Many key enzymes involved in AA metabolic network are responsible for generating inflammation mediators. The AA metabolic network with a multi-cellular ensemble of human polymorphonuclear leukocytes (PMNs), endothelial (EC) and platelet (PLT) cells. Extensive researches on the metabolism of AA metabolic network in human PMNs have been performed. Leukotrienes (LTs) LTs are the major inflammatory mediators produced in PMN [25]. In this paper, we study the dynamic properties of the AA metabolic network in human PMNs, as shown Fig. 1, to gain more insights into anti-inflammatory drug target design. Ordinary differential equations (ODEs) are constructed to simulate the its dynamics, where 24 equations were constructed (see Additional file 1). All the parameters of the ODEs are shown in Additional file 2.

### Determining the sensitive and insensitive individual drug targets in AA metabolic network

It is showed that LTs rather than prostaglandins (PGs) are the main inflammatory mediators produced in human PMNs [17]. Here, the disease state of the AA network is defined as a state where the output of LTs is markedly above the normal level. Since the standard parameters fitted correspond to the abnormal metabolism of LTs, the stable state under standard parameters is described as the disease state. The desired state after perturbation is defined as low output of inflammatory mediators, that is, the cumulative output of LTB4 should be smaller 10*%* than that in the disease state. Since it is difficult to give a definite cutoff to distinguish between normal and disease states and the threshold is set to be 10%. Eight enzymes in the AA metabolic network are selected as drug targets, because perturbing them individually can induce the transition of the AA metabolic network from a disease state to a normal one [3, 17].

The process of determining the sensitive and insensitive individual drug targets in AA metabolic network includes the following steps:

Step 1: Define the disease and normal states. The normal state is defined as a state where 1h cumulative production of LTB4 is less than 10% of that in the disease state. The fluxes of other metabolites are not monitored.

Step 2: Eight enzymes are chosen as drug targets (as shown in Table 3). We mark the initial concentration of the *i*-th enzyme with *E*_*di*_.

**Table 3:** Ranking the value of $\frac {\vartriangle E_{i}}{E_{di}}$ from small to large

Number	Enzyme	△*E*_*i*_/*E*_*di*_
1	CYP4F3	0.0769
2	PLA2	0.1979
3	PHGPx	0.213043
4	TXAS	0.99999
5	15-LOX	43.6388
6	5-LOX	135.4
7	LTA4H	15066606.44
8	12-LOX	1.52×10^17^

Step 3: Change the concentration of each target enzyme until the 1h cumulative production of LTB4 is just below 10% of that in the disease state. After 1h, we mark the changed concentration of the *i*-th enzyme with *E*_*hi*_.

Step 4: Define the degree of change in concentration of each enzyme as △*E*_*i*_/*E*_*di*_, where △*E*_*i*_=|*E*_*di*_−*E*_*hi*_|, and rank the value of △*E*_*i*_/*E*_*di*_ according to the degree.

To ensure the sensitivity of drug targets, we set *a*=1, i.e, when the transition is realized, the perturbation quantity is equal to or less than the initial concentration of the selected target. Then, four enzymes which satisfy △*E*_*i*_/*E*_*di*_≤1 can be selected as sensitive drug targets and the remaining four enzymes are insensitive, as shown in Table 3.

### Screening synergistic drug target combinations in AA metabolic network

#### Identifying the synergistic combinations of sensitive drug targets

**Fig. 2:**
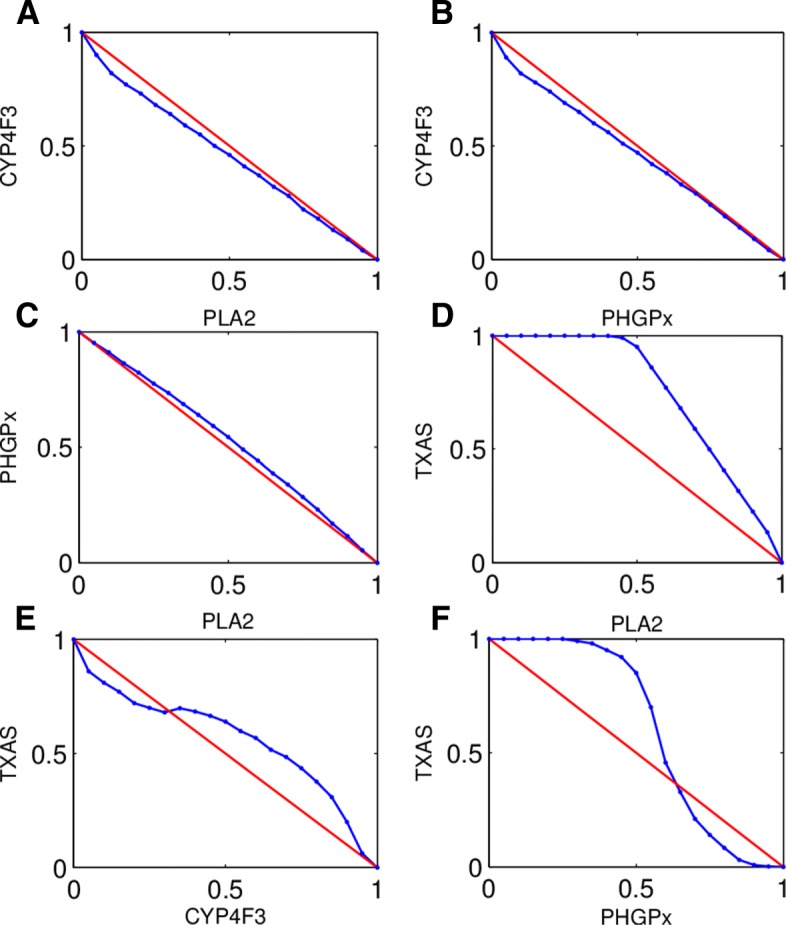
The result of identifying the synergistic and antagonistic combinations of sensitive drug targets. In this figure, there are three type curves in this figure. The points represent different drug doses. **a** and **b** are the synergistic combinations. **c** and **d** are antagonism combinations. **e** and **f** are hybrid type

We use CI to identify the synergistic and antagonistic combinations of sensitive drug targets. According to Eq. 2, we calculate CI value of every combination of sensitive drug targets. We mainly concern about the qualitative shape of the CI isobolograms for correctly identifying synergistic drug pairs, and use the smallest or largest CI of all dose combinations as the CI for one drug target pair. The CI isobolograms of each combination of sensitive drug targets are shown in Fig. 2.

We rank the combinations of sensitive drug targets by the value of CI and the degrees of synergism or antagonism according to Table 2, as shown in Table 4. It is obviously that there are four pairs of combinations that have synergistic effects, and three pairs of combinations have antagonistic effects. Especially, the combination of TXAS and CYP4F3 is the type of hybrid. For different drug dose combinations, TXAS and CYP4F3 may have synergy or antagonism.

**Table 4:** Description and symbol of synergy or antagonism in drug target combination of sensitive drug targets studies analyzed by CI method

Combination name	Value of Combination Index	Description	Graded Symbols
PHGPx-TXAS	0.8806	Slight synergism	+
TXAS-CYP4F3	0.91	Nearly additive	+ -
PLA2-CYP4F3	0.92	Nearly additive	+ -
PHGPx-CYP4F3	0.92	Nearly additive	+ -
PLA2-PHGPx	1.0442	Nearly additive	+ -
TXAS-CYP4F3	1.1850	Slight antagonism	-
PLA2-TXAS	1.45	Antagonism	- - -

#### Identifying the synergistic combinations of insensitive targets

**Fig. 3:**
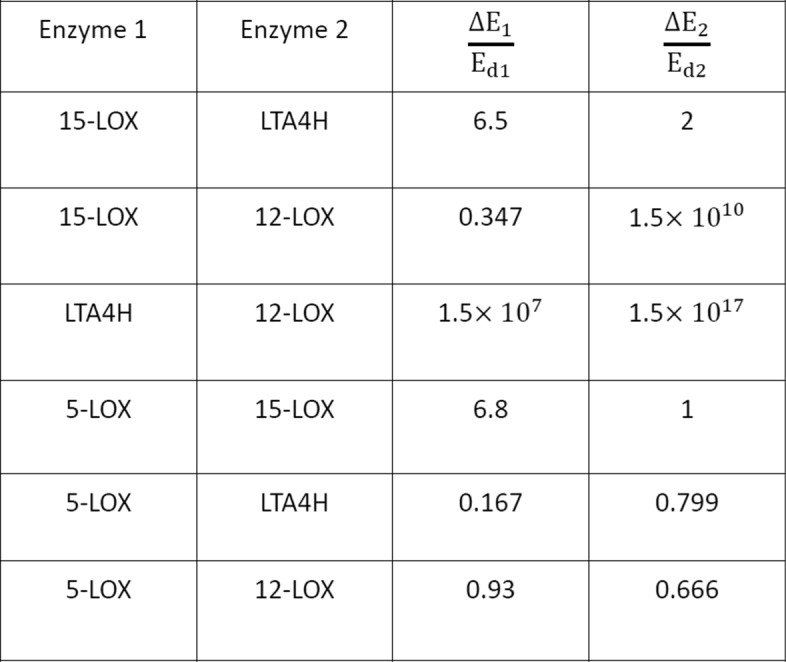
The result of calculating the △*E*_*i*_/*E*_*di*_ of insensitive drug targets. Two combination (5-LOX, LTA4H and 5-LOX, 12-LOX) can satisfy △*E*_*i*_/*E*_*di*_≤1

There are four insensitive drug targets which satisfy △*E*_*i*_/*E*_*di*_>1. Now, we combine them in pairs and calculate the value of △*E*_*i*_/*E*_*di*_ after combination, as shown in Fig. 3. It is obviously that two combinations satisfy the condition △*E*_*i*_/*E*_*di*_≤1. The two combinations are 5-LOX, LTA4H and 5-LOX, 12-LOX. According to our strategy, these two combinations are synergistic drug target combinations. In other words, these two pair targets are synergistic combinations and we can perturb them by drugs in a combinatorial way to realize the transition from the disease to the normal state. The result is partially consistent with the biological findings because 5-LOX is a standard drug target among the key enzymes involved in the network responsible for generating inflammation mediators [17]. More importantly, some studies also support that the two combinations of 5-LOX, LTA4H and 5-LOX, 12-LOX are feasible drug target combinations in AA metabolic network [26, 27].

#### Identifying the synergistic combinations of sensitive and insensitive drug targets

We have determined that four among all eight drug targets are sensitive and the other four are insensitive. So, there are 16 pairs of combinations of sensitive and insensitive drug targets totally. For the sensitive drug targets, we set *α*=0.8 and *α* is the constant in formula (3). In other words, the quantity of perturbation or drug to the sensitive target needed can be decreased 20% after the auxiliary perturbation to the insensitive target is performed. More exactly, synergistic drug target combination means △*E*_*i*_/*E*_*di*_≤1 for the insensitive drug target and (△*E*_*j*_)^1^/(△*E*_*j*_)^2^=0.8 for the sensitive drug target.

**Fig. 4:**
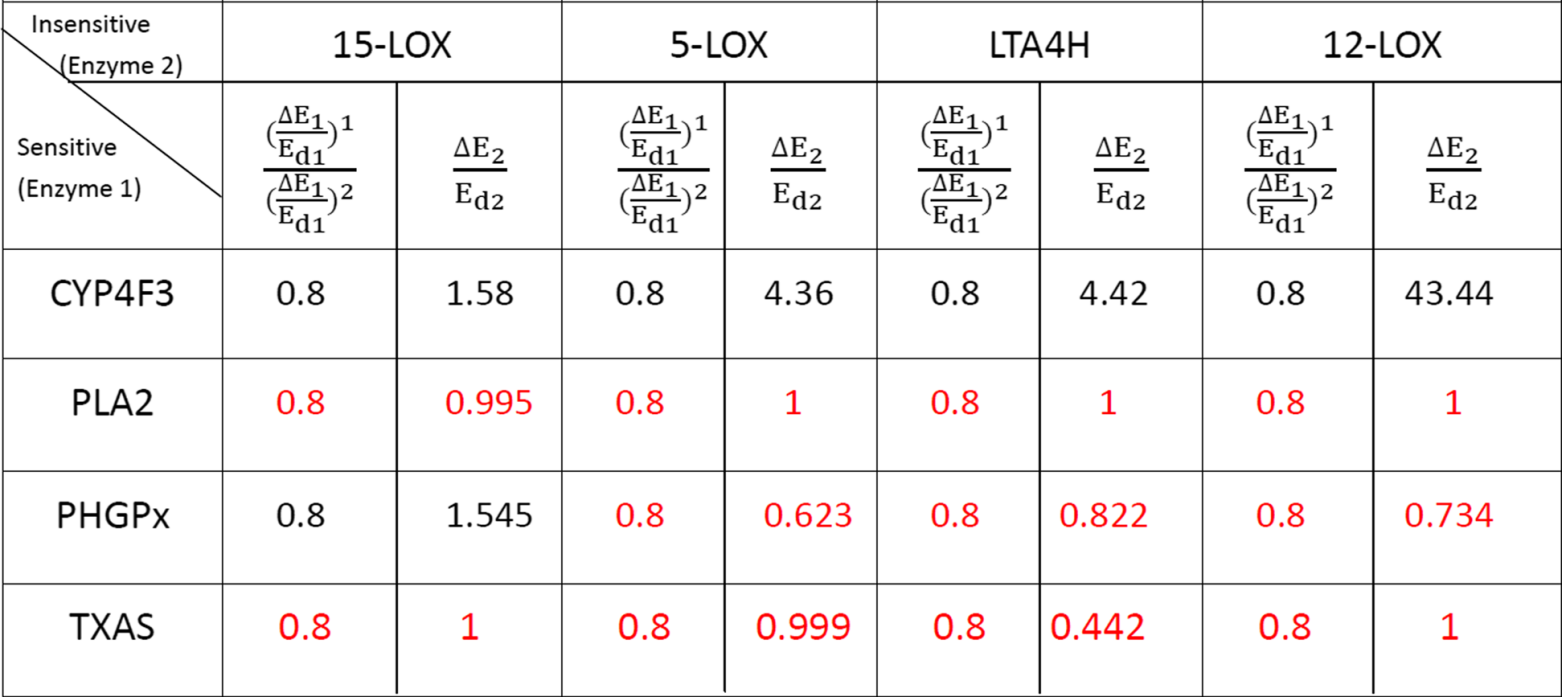
The combination of sensitive drug targets and insensitive drug targets. The red numbers represent the synergistic combinations

After calculating, there are 12 pairs of drug target combinations which can satisfy above conditions, as shown in Fig. 4. There are thee combinations have been proven to be viable by the corresponding biological experiments, including the combination of 15-LOX and PLA2 [28], combination of PLA2 and LTA4H [29], and combination of PLA2 and 5-LOX [30]. Especially, it has been found that pathway including PLA2 and LTA4H in AA metabolic network is very important for traditional Chinese medicine anti-inflammatory herbal formulae and the treatment of cancers [29, 31, 32].

## Discussion

Due to the complexity nature of many diseases and the rising drug resistance, drug combination is becoming the standard treatment of many complex diseases. In this paper, we present a strategy to identify effective drug target combinations. Different from existing methods, the proposed strategy aims to identify effective drug target combinations from the perspective of network or systems biology. First, we select the enzymes as individual drug targets which can induce a transition from a disease state to a desired state when they are perturbed alone. Then, we divide them into sensitive and insensitive drug targets. Finally, we identify the synergistic and antagonistic combinations of all two types drug targets.

For the sensitive drug targets, we judge the synergistic and antagonistic of combination by the method of CI. For the combination of insensitive drug targets, two pairs of combination can restore the network to the normal state under our condition, and these combinations may be of great significance in biomedicine. For example, in the processing of disease treatment, when two drugs are used alone, they can not cure the disease, but when the two drugs are used simultaneously, the disease can be cured or the effect can be significantly improved. For the combination of sensitive and insensitive drug targets, it can reveal the intrinsic mechanism of the treatment of some diseases. For example, a single drug interventions for a disease have reduced or no effect due to the rapid drug resistance response, however the joint intervention of two or more drugs show better efficacy and lower drug resistance. Similar situation can be often seen in the treatment of diseases. In many cases, although a single drug can cure a disease, the simultaneous intervention of multiple drugs not only can increase the efficacy but also can reduce the side effects of drugs.

**Fig. 5:**
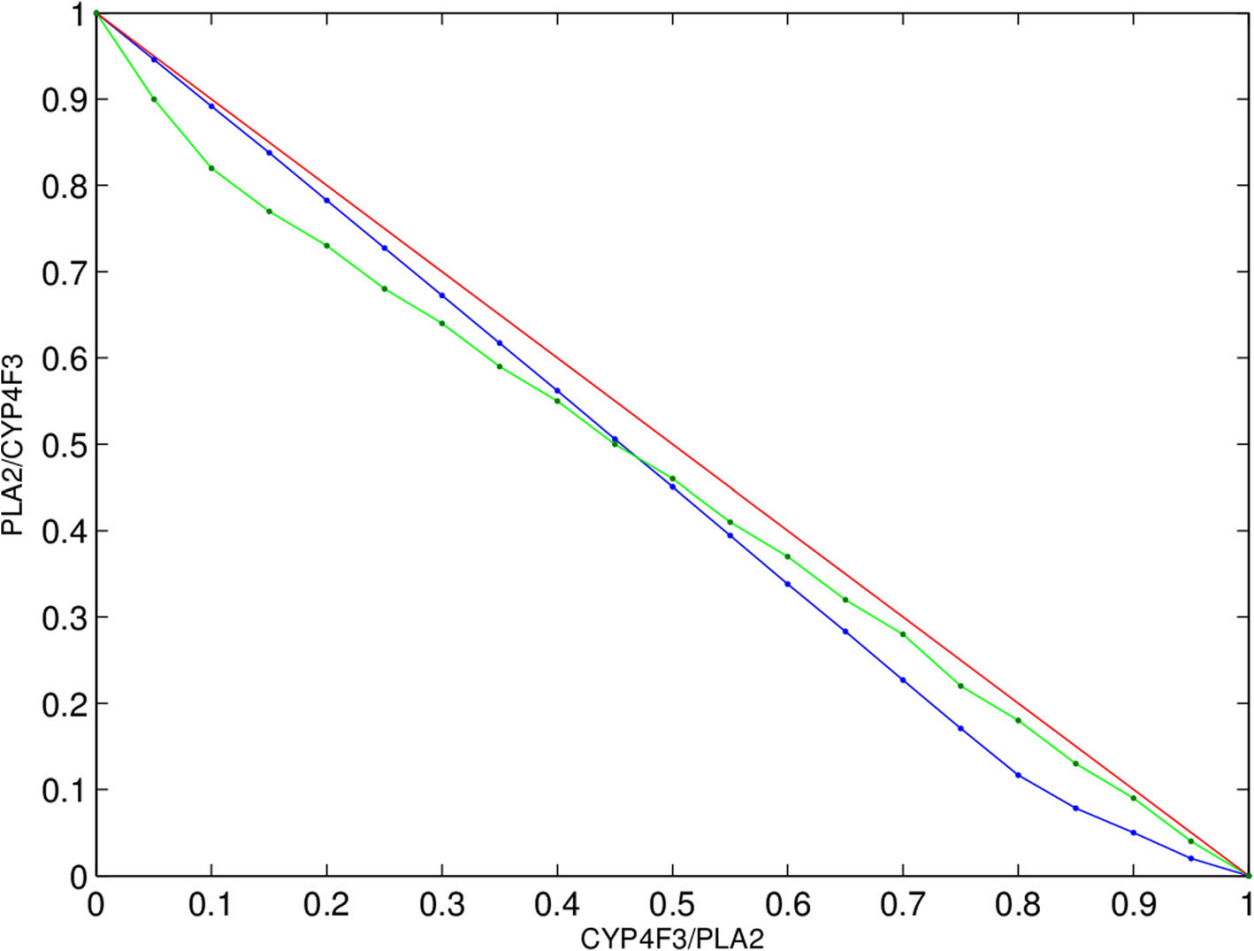
The CI isobolograms of PLA2 and CYP4F3

In the part of identifying the synergistic and antagonistic combinations of the sensitive drug targets by computing CI, for any one pair of combination, we mark one drug target with A and another with B. In the process of calculating CI, we first keep the concentration of A unchanged, and then adjust the concentration of B to restore the disease network to a normal state. Then we keep the concentration of B unchanged and adjust the concentration of A to restore the disease network to normal. We can get the same result in the two methods. We give one example of the combination of PLA2 and CYP4F3 in Fig. 5.

## Conclusion

In this paper, we provide a new strategy to find the potential effective drug target combinations of all three types of drug target combinations involved in our paper. Among the combinations of drug targets we have screened, there are five combinations have been proven to be viable by the corresponding biological experiments, including the combination of 5-LOX and 12-LOX [26], combination of 5-LOX and LTA4H [27], combination of 15-LOX and PLA2 [28], combination of PLA2 and LTA4H [29, 31, 32] and combination of PLA2 and 5-LOX [30]. What’s more, if we can combine the price and doses of drugs, we can have a better optimization. Although we mainly apply the proposed strategy to screen the effective combinations of two drug targets, the strategy can also be used to screen effective combinations of multiple drug targets in a straightforward manner.

## Methods

### Construction of the AA metabolic network model in human PMN

On the basis of the AA metabolic network, a set of ODEs were constructed to describe cell behavior of inflammation in human PMN(see details in Supplementary information). The ode 45 s routine of Matlab was used to integrate the ODEs. Michaelis Menten equations (Eq.3) were used to describe enzyme catalytic reactions in the network:

4 $$ \frac{d[S]}{dt}=\frac{K_{cat}[E_{t}][S]}{K_{m}+[S]}  $$

where [*S*] is the concentration of the substrate, [*E*_*t*_] is the total concentration of enzyme, *K*_*cat*_ is turnover number, and *K*_*m*_ is the Michaelis-Menten constant.If competitive reversible inhibitors are involved in the catalysis, the equation is:

5 $$ \frac{d[S]}{dt}=\frac{K_{cat}[E_{t}][S]}{K_{m}(1+\frac{[I]}{K_{i}})+[S]}  $$

where [*I*] is the concentration of inhibitor and *K*_*i*_ is the inhibition constant, which is defined as:

6 $$ K_{i}=\frac{[E][I]}{[EI]}  $$

If the inhibitors are irreversible, we assume the enzymes would decay according to the following equation:

7 $$ \frac{d[E]}{dt}=-K[E][I]  $$

where K is a constant.When activators are involved in the catalysis, we use the following equation:

8 $$ \frac{d[S]}{dt}=\frac{K_{cat}(1+([A]/KI))[E_{t}][S]}{K_{m}+[S]}  $$

where [*A*] is the concentration of activator and *KI* is a constant.When up regulation occurred though transcription, we described its effect with the following equation:

9 $$ \frac{d[E]}{dt}=\frac{k[g]^{2}}{[g]^{2}+[k]^{2}}  $$

where [*g*] is the concentration of the metabolite up regulating the transcription of the enzyme, *K* and *k* are constants.

## Additional files

Additional file 1 The ordinary differential equations of AA metabolic network was developed. A series of ODEs was established to simulate unicellular behavior, which included 24 initial concentrations and 45 reaction constants. (PDF 130 kb)

Additional file 2 A total of 23 reaction constants was taken from experimental values, while the others were obtained by fitting the calculated production of LTB4. (PDF 119 kb)

## Abbreviations

AA: Arachidonic acid
CI: Combination index
EC: Endothelial
LTs: Leukotrienes
ODEs: Ordinary differential equations
PGs: Prostaglandins
PLT: Platelet
PMNs: Human polymorphonuclear leukocytes
TCM: Traditional Chinese medicines
