# Supplementary material for: Screening drug target combinations in disease-related molecular networks
Source: BMC Bioinformatics. 2019 May 1;20(Suppl 7):198. doi: 10.1186/s12859-019-2730-8 (PMC6509874; doi:10.1186/s12859-019-2730-8)
Supplement: Supplementary file 2 — A total of 23 reaction constants was taken from experimental values, while the others were obtained by fitting the calculated production of LTB4. (PDF 119 kb) [file 12859_2019_2730_MOESM2_ESM.pdf]

The corresponding parameters of AA metabolic network

December 29, 2018

A total of 23 reaction constants (as shown in additional file 1) was taken from experimental values, while the others were obtained by fitting the calculated production of LTB4. In addition, the corresponding parameters are shown in Table 1, 2, 3.

Table 1. The corresponding parameter derived from parameter fitting.

parameter	value
$Ki_{AA \rightarrow PGES}$	0.3
$Ki_{15-HETE \rightarrow PGES}$	0.53
$Ki_{PGE2 \rightarrow COX2}$	2.8
$Ki_{15-HPETE \rightarrow TXAS}$	0.52
$Ki_{PHG2 \rightarrow TXAS}$	0.029
$Ki_{15-HPETE \rightarrow 5-LOX}$	0.01
$Ki_{12-HETE \rightarrow 5-LOX}$	30
$Ki_{15-HETE \rightarrow 5-LOX}$	4
$Ki_{LTA4 \rightarrow 5-LOX}$	0.175
$Ki_{5-HPETE \rightarrow 5-LOX}$	0.26
$Ki_{PGE2 \rightarrow 5-LOX}$	72
$Ki_{5-HETE \rightarrow 5-LOX}$	6.3
$Ki_{LTA4 \rightarrow LTA4H}$	129
$Ki_{12-HETE \rightarrow CYP4F3}$	0.29
$Ki_{5-HETE \rightarrow CYPEF3}$	0.8
$Ki_{15-HETE \rightarrow 12-LOX}$	1.5
$Ki_{15-HPETE \rightarrow 12-LOX}$	0.23
$Ki_{12-HPETE \rightarrow 12-LOX}$	1.6
$Ki_{12-HPETE \rightarrow PLA2}$	260
$Ki_{15-HPETE \rightarrow PLA2}$	430
$Ki_{LTB4 \rightarrow PLA2}$	180
$Ki_{5-HETE \rightarrow PLA2}$	240
$Ki_{LTB4 \rightarrow 5-LOX}$	0.022
$Ki_{PGE2 \rightarrow 15-LOX}$	0.15
$Ki_{PGE2 \rightarrow 15-LOX}$	0.000023
Ki	500

Table 2. The K_m and K_{cat} of enzymes used in the ODEs and the corresponding parameter has no direct value from experiments and is derived from parameter fitting.

enzyme	K_{cat}	K_m
$15-LOX$	5000	13
$5-LOX$	6000	1.4
$LTA4H$	150	20
$CYP4F3A$	150	3.9
$PHGPx$	2000	58
$COX-2$	1000	33
$PGES$	3000	160
$TXAS$	1599	4
$PLA2$	3600	2600
$12-LOX$	9500	160

Table 3. The decay rate of molecules used in ODEs and the corresponding parameter has no direct value from experiments and is derived from parameter fitting.

parameter	value
$Kd_{15-HPETE}$	0.36
$Kd_{15-HETE}$	1
Kd_{TXA}	0.0001
Kd_{TXB}	0.0013
Kd_{5-HETE}	0.00042
Kd_{LTA4}	0.0017
Kd_{LTB4}	0.00063
Kd_{15-LOX}	0.00083
Kd_{exoAA}	0.017
Kd_{AA}	0.14
